# Supplementary material for: Splice-Junction-Based Mapping of Alternative Isoforms in the Human Proteome
Source: Cell Rep. Author manuscript; Available in PMC 2020 Jan 15. (PMC6961840; doi:10.1016/j.celrep.2019.11.026)
Supplement: 2 [file NIHMS1546469-supplement-2.zip › DF1/PXD006675/RightVentricle/RightVentricle_23_PPP1R1A_VEHHLGQQQQGEEPEGAAESTGTQESRPPGIPDTEVESR.pdf]

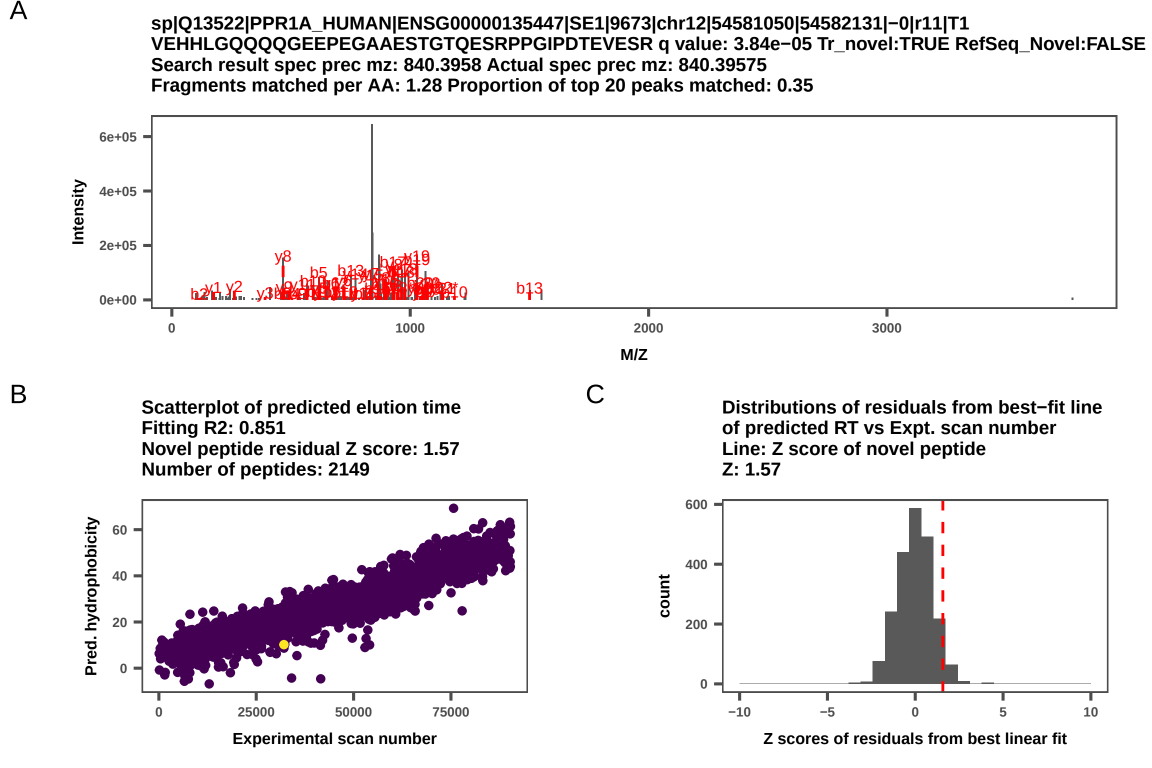
A
sp|Q13522|PPR1A_HUMAN|ENSG00000135447|SE1|9673|chr12|54581050|54582131|−0|r11|T1
VEHHLGQQQQGEEPEGAAESTGTQESRPPGIPDTEVESR q value: 3.84e−05 Tr_novel:TRUE RefSeq_Novel:FALSE
Search result spec prec mz: 840.3958 Actual spec prec mz: 840.39575
Fragments matched per AA: 1.28 Proportion of top 20 peaks matched: 0.35
Intensity
6e+05
4e+05
2e+05
0e+00
y8
b5
b13
b2
y1
y2
b13
0
1000
2000
3000
M/Z
B
Scatterplot of predicted elution time
Fitting R2: 0.851
Novel peptide residual Z score: 1.57
Number of peptides: 2149
Pred. hydrophobicity
60
40
20
0
0
25000
50000
75000
Experimental scan number
C
Distributions of residuals from best−fit line
of predicted RT vs Expt. scan number
Line: Z score of novel peptide
Z: 1.57
count
600
400
200
0
−10
−5
0
5
10
Z scores of residuals from best linear fit
